# Dialogue is a prerequisite for the nurse–patient relationship in nutritional care: A secondary analysis using the fundamentals of care framework

**DOI:** 10.1111/scs.13094

**Published:** 2022-07-01

**Authors:** Pia Søe Jensen, Vibeke Nørholm, Ingrid Poulsen, Helle Vendel Petersen

**Affiliations:** ^1^ Department of Clinical Research Copenhagen University Hospital Hvidovre Denmark; ^2^ Department of Orthopaedic Surgery Copenhagen University Hospital Hvidovre Denmark; ^3^ Research Unit of Nursing and Health Care Health, Aarhus University Aarhus Denmark; ^4^ Department of Brain Injury Copenhagen University Hospital Rigshospitalet Copenhagen Denmark

**Keywords:** dialogue, fundamentals of care, nurse‐patients relationship, nutritional care, orthopaedic care, qualitative research, secondary analysis

## Abstract

**Background:**

Providing good nutritional care is complex as it goes beyond assessing and ensuring the patients' dietary needs. So far, nutritional research has mainly focused on establishing evidence for the nutritional treatment, while less attention has been on the complexity of providing nutritional care. The Fundamentals of Care (FoC) describes five elements (focus, knowledge, anticipate, evaluate and trust) essential for establishing a nurse–patient relationship as a foundation for quality care. By studying how these elements shape nutritional care and dialogue, we can explore and describe the complexity of nutritional care.

**Aim:**

By using the FoC framework as an analytic framework, this study explores how the nurse–patient relationship shapes the nutritional care of orthopaedic patients.

**Method:**

This study is a secondary analysis using deductive content analysis of interviews with patients undergoing major orthopaedic surgery, nursing staff and observations of interactions between nursing staff and patients. The core dimension of the FoC framework, ‘Establishment of relationship,’ was used as an analytic framework.

**Result:**

The nurses perceived serving meals and providing nutritional supplements as an essential part of the nutritional care. Still, the nutritional care was organised as a routine task to be less time‐consuming. Appropriate care was initiated when the nursing staff explored patients´ food preferences. When the nursing staff failed to familiarise themselves with the patient's preferences, the patients interpreted nutritional care as unrelated to their needs, resulting in a lack of trust.

**Conclusion:**

The need for efficiency within nutritional care must not compromise the patients' need for dialogue with the nurse. Establishing a trusting relationship between nurses and patients prevents nutritional care from becoming a routine task unrelated to the patients' needs.

## INTRODUCTION

Caring for malnourished patients can be challenging [1] as nutritional problems are often complex and go beyond dietary intake [2]. The prevalence of malnutrition is between 20% and 50% amongst hospitalised patients +65 years of age [3, 4, 5]. These numbers also apply to older orthopaedic patients [6, 7]. Malnutrition increases the risk of muscle loss and loss of motor independence in older patients [8], leading to prolonged hospitalisation, medical complications, higher healthcare cost and increased mortality [6, 7]. The high prevalence of malnutrition underscores the need for nurses to improve nutritional care and overcome barriers to providing adequate nutritional care. Barriers like low priority, inflexible food service and limited multidisciplinary support affect how nurses provide nutritional care [9, 10].

Nutritional care has always been an essential part of nursing [11]. Evidence‐based nutritional treatment and care guidelines are available [12, 13]. According to the guidelines, nutritional care includes: screening for nutritional risk, providing an individual nutritional plan, managing the nutritional therapy, monitoring the dietary intake and evaluating and adjusting the nutritional plan [12]. However, nutritional care goes beyond ensuring the patients' dietary needs and becomes complex when nurses adjust dietary recommendations to fit patients' individual preferences and needs [14]. So far, nutritional research has mainly focused on establishing evidence for the nutritional treatment strategies, while less attention has been on the complexity of nursing in providing nutritional care [15].

Identifying needs and problems underlying malnutrition requires competencies within both nursing and nutrition [16, 17, 18]. While patients value and call for dialogue with nurses as a part of their care [18, 19, 20], nurses may tend to independently steer clinical decisions concerning nutritional care without including the patients' preferences [21]. Limited time, resources and lack of knowledge and awareness amongst the nurses may stand in the way of integrating individualised nutritional care into daily practice [16, 18, 21]. When nurses fail to initiate a dialogue, they overlook their patients' nutritional problems [22], resulting in patients feeling unnoticed with unimportant dietary problems [23]. These issues highlight the need for nurse–patient dialogue [18, 19, 20] and emphasise person‐centred care as a foundation for nutritional care. The core elements in person‐centred care are patient participation and involvement, the relationship between the patient and the nurses and the context of care [24]. McCormack suggests that nurses move beyond a focus on technical competence and engage in person‐centred nursing, which offers a way of reinstating the value of the fundamentals of nursing care [25, 26]. Even though nurses recognise the importance of nutritional care, patients still receive inadequate nutritional care [9, 21].

The Fundamental of Care (FoC) framework focuses on the patients' essential needs to ensure their physical and psychosocial wellbeing. A core dimension of any nursing action is to establish a positive and trusting nurse–patient relationship [27, 28]. The FoC framework explains how each of the following five elements influences the nurses‐patient relationship:[27]

*developing trust with the patient, focusing and giving the patients undivided attention, anticipating their needs, knowing enough about the patient to act appropriately and evaluating the quality of the relationship*

To explore how nurses provide nutritional care, we propose an interpretation of the nutritional care that applies to each of the five elements from the ‘Establishing the relationship’ as an analytic framework. By focusing on how nurses communicate and interact to establish a relationship with the patients, we can explore elements of importance within nutritional care.

## AIM

This study explores how the nurse–patient relationship shapes nutritional care for patients undergoing acute orthopaedic surgery.

## METHOD

### Design

The study is a qualitative secondary deductive analysis of data from single‐ and focus‐group interviews with patients and nurses collected at an orthopaedic ward. The data originate from two previous studies on patients' and nurses' perspectives on nutritional care [21, 23]. We aimed to gain further insight into establishing the nurse–patient relationship in a clinical setting by including observational data. The combined data represents different research methods and aspects of nutritional care at different time points. To identify meaning units of relevance for establishing the nurse–patient relationship and how the dialogue shapes nutritional care, we defined the five elements from ‘Establishing the relationship’ from the FoC framework: *focusing, knowing, trusting, anticipating* and *evaluating* to comply with nutritional care [24]. See Table 1. The COREQ (consolidated criteria for reporting qualitative research) criteria were used to ensure a comprehensive reporting of the study [29].

**TABLE 1:** Description of the nutritional care and research questions according to the five elements from ‘Establishing the relationship’ in the FoC framework

Elements of FoC	The original description from the FoC framework^a^	The proposed description of good nutritional care	Transformed research questions
**Focusing** *How do I give you my undivided focus/attention?*	The nurse develops skills of focusing on the patient in real‐time without being distracted. Focusing may only involve very short bursts of time but are hugely important in terms of surveillance, anticipation, detecting changes in patient state	Nurses give attention to patients' nutritional preferences and initiate a dialogue either about or related to patients' nutritional issues	How does the nurse describe giving undivided focus/ attention to the patients? How do patients experience undivided focus/attention from the nurses? How does undivided focus/attention appear in clinical practice?
**Knowing** *What do I need to know about you and why you are here*?	The nurse must balance the need to know information with the patient's sense of control, privacy and dignity. Having the patient repeat the same information to numerous staff is disrespectful to the individual patient	Nurses integrate knowledge about the consequence of low dietary intake, the effectiveness of nutritional care interventions and information about the patient nutritional status into the care	How/Do nurses describe/value/use their knowledge about the patients? How do patients' experience/notes when nurses know about them? How do nurses show they use their knowledge?
**Anticipating** *How best can I help guide you on this journey?*	The nurse, by asking this question, will be able to consider the proposed course of action and start discussing this with the patient as part of the recovery process	Nurses must promote and support the patients physical, mental or emotional ability to eat into the care to fulfil patients' nutritional needs.	How/Do nurses describe/value the ability to anticipate patients' needs? How do patients describe/experience when nurses anticipate the course of actions? How do nurses show their ability to anticipate the patient's recovery process?
**Evaluation** *How will we know* *it is working?*	Both the patient and the nurse should continuously review progress and give feedback to each other on how things are going. The patient and the nurse also negotiate who else needs to be involved in this review process (e.g. relative or carer)	Nurses include the patient's views or experience and use relevant and available information to evaluate the fulfilment of patients' nutritional needs.	How/Do nurses describe how they evaluate the care? How do patients' experience/notes when nurses evaluate their process? How do nurses evaluate the care/ patient's progress?
**Trusting** *How can we develop a trusting relationship*?	There may be numerous staff caring for the individual patient, so how does trust between two people become established if the encounters are short, intermittent and infrequent?	Nurses incorporate patients views and experiences into the care and show through verbal communication or by actions the intention to engage in nutritional care and to involve the patient	How/Do nurses describe being/creating trustworthy? How do patients describe trust in nurses? How do nurses show trustworthy behaviour?

Kitson [27]

### Participants and setting

Data were collected at the Amputation and Wound unit and the Hip Fracture unit at the Department of Orthopaedic Surgery in a University hospital in the urban site of Copenhagen. Patients undergoing amputation are characterised by multimorbidity, polypharmacy, low health literacy and socioeconomic status and are most prevalent amongst men with an average age of 68 years, while women tend to be approximately 10 years older [30]. Patients with a hip fracture are also characterised by high age, multimorbidity and frailty [31]. Both patient groups are at high risk of reduced mobility and increased mortality post‐surgery. The nursing staff were trained in pre‐and post‐surgery care [31, 32]. This study includes nursing staff with work experience ranging from very experienced to newly qualified nurses, nursing assistants and nurse students. Thirteen nurses from the amputation unit participated in the four focus‐group interviews, and 17 patients who had undergone amputation participated in single interviews. Nursing staff from both units participated in the 115 nurse–patient interactions. For this study, the ‘nurse–patient relationship’ refers to the relationship between patients and nursing staff.

### The organisation of nutritional care

The hospital offered a lá carte menu for all patients with several breakfast, lunch and dinner options. The nurses were responsible for supporting the patients in selecting dishes that fulfilled their individual nutritional needs. If a patient could not participate, for example due to physical or cognitive problems, nurses selected the dishes based on their knowledge of patients' preferences. The hospital kitchen delivered the designated meals to each patient on a tray. The nursing staff provided drinks (water, milk or nutritional supplements) when serving the food trays. All meals were served at small tables in the hallway or by the patient's bedside. According to the department's strategy for nutritional therapy, the patient's dietary intake was monitored for 4 days’ post‐surgery.

### Data collection

The first and last author performed the interviews at the department of orthopaedic surgery.

The four focus‐group interviews with 13 nurses were performed to identify nurses' perceptions of barriers to nutritional care and lasted between 72–100 min. The interview guide was designed according to the Theoretical Domains Framework [33], and a purposive sampling strategy included nurses from 4 months to 30 years of work experience. All original interviews were transcribed and anonymised for this study. More details have been published [21, 23].

The patient interviews were collected as semi‐structured single face‐to‐face interviews to examine patients' perspectives on food preferences, dietary counselling and nutritional care following lower extremity amputation. All patients were diagnosed with diabetes or atherosclerosis. The interview guide explored three topics; patient perspective on daily food, experience with the hospital's nutritional care and expectations of food habits following discharge. The participants had a median age of 72 years (46–78 years) and included six women. The interview lasted a median of 43 minutes. (23–65 minutes).

The observational data contain field notes from 23 hours of observations of 115 patient interactions with 48 nursing staff members. The purpose was to explore the nurse–patient dialogue related to nutritional care. The interactions were observed during three care situations: (1) Nursing staff assisting patients ordering from the a la carte menu; (2) Nursing staff serving a meal to the patient; (3) Nursing staffs´ collecting the patients' food tray. The observations were performed using a partly participating approach [34] and carried out in both units over 2 weeks at lunchtime by two bachelor nurse students in their final year, supervised by the first author. The students positioned themselves as students who followed the nursing staff. Field notes were transcribed daily, followed by a more comprehensive description of the observations.

### Analysis

The three data sets were anonymised, imported into Excel spreadsheets and deductively analysed using content analysis inspired by Elo & Kyngäs [35]. Prior to the analysis, a structured analysis matrix was developed based on the five elements of ‘Establishing the relationship’. To include the nutritional aspect, we propose a new interpretation of the original FoC description of each element (Table 1). To operationalise the matrix, we transformed the original FoC framework questions related to the five‐element from, for example ‘How do I give you my undivided focus/attention? [24]‘ to ‘How do nurses provide/use/show..?’ and ‘How do patients respond/express..?’, (Table 1).

**FIGURE 1:**
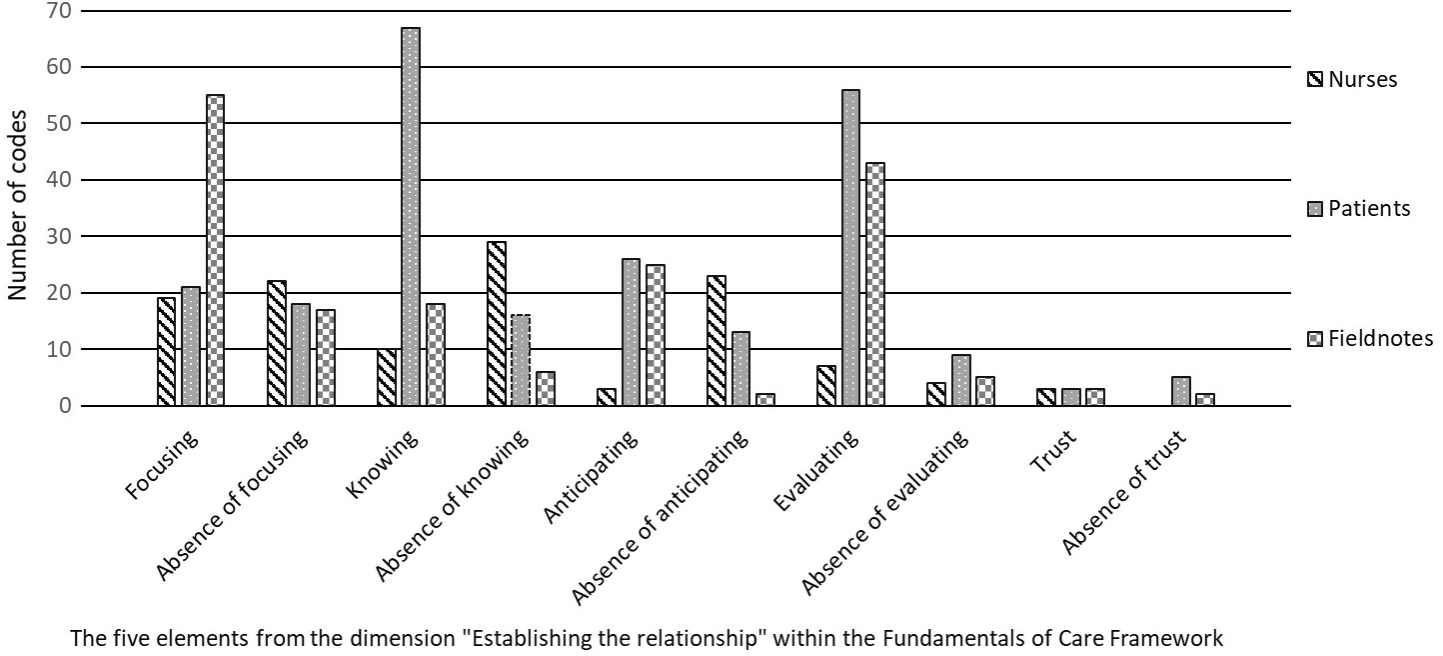
Distribution of the codes from interviews and fieldnotes according to the presence or absence of each elements/The five elements from the dimension "Establishing the relationship" within the Fundamentals of Care Framework

The three datasets were thoroughly read and analysed separately using the matrix. First, we identified sections of the original text in the three data sets relevant to this study. These sections were merged into three new datasets. The text was condensed and coded according to the predefined categories in the matrix (Table 2). The second and last authors conducted the coding separately and discussed it until a consensus was reached. Codes describing the presence or absence of each element were included (Figure 1). A consensus was reached amongst all authors to form the final version of the matrix to validate the analysis. All authors are experienced researchers within the field of qualitative research.

**TABLE 2:** The matrix of condensed meaning units represents the presence and absence of each aspect of commitment to care

	Focusing	Knowing	Anticipating	Evaluating	Trusting
Nurse interview	‘Observe and talk to the patient about how he is doing.’	‘Provide the right diet, and see changes in the patient.’	‘Includes the patient's preferences and seek to fulfil these.’	‘Adjusts the diet according to what the patient can manage.’	‘Took the time to sit with the patient while he was eating.’
	‘Focus is on dietary monitoring and less on the patient's nutritional needs.’	‘No time to familiarise with the patient to be able to assess the patient's nutritional needs’	‘It's up to each nurse to assess and decide whether the patient gets enough to eat.’	‘It's a problem that we help each other to collect the meal trays if you need to focus on what the patient eats.’	‘Do not have the time to make a relationship with the patient during meal serving.’
Patient interview	‘Feels that the nurses have time for talks and to guide me.’	‘The patient became aware that he wasn't eating enough when the nurses started to ask.’	‘ There were facts about food the patient would not know about if the nurses had not talked about it.’	‘Options can motivate you to try and eat a bit more.’	‘Assuming the food was good, he was encouraged to eat.’
	‘Could well have wished that a nurse had sat down and explained why the requirement to drink is so high.’	‘Lacked the blood glucose measurement result and became unsure about the insulin.’	‘ No one has explained to the patient why protein is good for her.’	‘Nobody talked about his diet, and he needed food he could eat rather than being forced to choose from the menu card.’	‘The nurse was very insistent that he should take insulin before the meal, but he knows what he needs.’
Fieldnotes /observations	‘Informs the patient about the importance of consuming protein for the wound to heal.’	‘The patient is offered a protein drink because he does not eat enough. He gets one with a taste he likes.’	‘The nurse negotiates with the patient and makes her order a little more dessert and fruit.’	‘The patient has not eaten enough; however, the calories are good. The nurse asks if the patient has nausea.’	‘Relatives are informed of the patient's dietary intake; the nurse offered soup as the patient has difficulty eating.’
	‘The patient is clearly ill and has difficulty breathing. The nurse continues to order food for the patient.’	‘The patient sleeps. He has not eaten very much; nobody talks to the patient about it.’	‘The patient is unsure about how much she should drink when told that she should drink a lot.’	‘The nurses take out meal trays without talking to the patients; one patient says he wants to keep his yoghurt.’	‘The nurse finish ordering the food on her screen and leaves the patient without asking how she is feeling.’

The codes in the grey row exhibit codes for the presence of each element, and the white exhibits codes for the absence.

### Ethics

According to the Helsinki Declaration, all participants received oral and written information about the purpose of the original studies. The participants were informed about anonymity, confidentiality and the possibility to withdraw at any time before participating. All participants signed a consent form agreeing on publications within the topic of nutritional care. The original studies were reviewed by the Ethical Committee of The Capital Region of Denmark (H‐3‐2014‐FSP67). According to Danish law, studies without invasive procedures do not require formal evaluation. The Danish Data Protection Agency approved the original studies (AHH‐2014‐041‐03395).

## RESULTS

The five elements essential for establishing a nurse–patient relationship were identified in all three datasets (Table 2). The results for each element are presented separately with the original FoC description [24], followed by a new proposed description focussing on nutritional care (Table 1). The results of each element are first presented from the nurses' perspectives, followed by the patients' and field observations. When relevant, citations are presented to illuminate the analysis.

### Focusing

Focusing is present when the nurses give their patients undivided attention during a care interaction and observe or detect changes in their condition. Focusing is not related to time or resources but to the ability to identify the need for care. [24]. Focus in nutritional care is when nurses provide undivided attention by incorporating the patients' nutritional preferences into the care, for example by initiating a nurse–patient relationship (Table 1).

Nurses expressed a wish to be more attentive to the patients' dietary needs and systematically incorporate patients' preferences, eating habits and data from the nutritional screening into the care. Appropriate care was initiated when nurses paid attention, for example dialogue exploring patients´ food preferences. The nurses described a gap between their wish to be attentive to the patients´ dietary needs and the obligation to prioritise tasks in a busy schedule.

The patient's perception of the nutritional care was determined by the nurses' ability to focus on the patient's nutritional issues, ranging from unnecessary to an integrated and essential part of the care. Some patients highlighted the importance of the nurse–patient dialogue as they perceived this to be essential for handling nutritional problems. The patients believed ‘the task of’ serving meals was unrelated to their nutritional care. The patients describe how the nursing staff could show attention to their nutritional care by reminding them to eat.

*And then there were the extra sip feeds. If they had been on my table for more than half an hour, someone would knock on the table: ‘Eat.’ I did not feel forced to do it, but it was a reminder for me. (Patient 13)*

When nurses explored the patients eating habits and dietary preferences, it highlighted the importance of nutritional care. Additionally, it increased the motivation for eating amongst the patients. The nurses' focus on nutrition made the patients feel less alone in dealing with their nutritional problems. When nurses showed attention to the patients' dietary needs, the patients responded positively by being motivated to eat.

*I have an inner motivation, but of course, I feel motivated (to eat, eds.) when they offer me something extra to eat. (Patient 17)*

Serving food was observed to be organised as a service task. The trays were transferred to two transportations waggons and were distributed to the patients according to the room number. For the most part, only two to three of the nursing staff participated. The nursing staff prioritised quickly distributing and collecting the patients' meal trays without interacting about nutritional issues.

*The nurse places a tray on the patient's bed table. The patient has difficulty getting the lid off the soup. Receive no assistance from the nurse. (field note, 2. observer)*

Sometimes the nursing staff offered nutritional alternatives during meal serving, but it was also observed that plates could be placed out of reaching distance without this being noticed by the nurses. When the nursing staff used the meal serving situation to explore the patients' nutritional needs, the focus was on the patients' food preferences, ability to eat or appetite level. When a more extended nurse–patient interaction was observed, the nurses engaged with the patients, for example about the diet content.

*The nurse brought lunch to the patient and placed the tray on the patient's bed table. They talk about the contents of the meal and how a high level of protein can increase the healing process of the patient's leg ulcer. (Field notes, 1. observer)*

### Knowing

Knowing is when nurses use knowledge about the patients' condition, background or preferences while balancing the need for information with the patient's sense of control, privacy and dignity [24]. Knowledge within nutritional care is when nurses gain knowledge about patients' nutritional state, including using available information to evaluate patients' nutritional issues, for example nutritional preference and integrating relevant information to improve the patients' nutritional status (Table 1).

The nurses did not routinely explore or document the patient's food preferences. Instead, they used available and easily accessible information from either the nursing or medical record to plan the patients' nutritional care. Consequently, the nursing staff made decisions about nutritional care without involving the patients.

*We take action based on what we see and what they need, the patients. We try to suggest and offer other types of food. (Focus group 3)*

The patients noted if the nursing staff has had limited insight into their nutritional status and condition. Some patients did not expect the nursing staff to know their eating habits or food preferences as they did not perceive nutrition as a part of their care or treatment. When patients were unable to care for themselves, they highly valued when nurses guided them and discussed possibilities of nutritional supplements.

The patients did not always interpret the nursing staff’ actions as linked to their nutritional issues. In contrast, the observations showed that the experienced nurses offered nutritional alternatives and explored the patient's preferences during meal serving more often than the newly qualified nurses or nurse students. When the nursing staff used their knowledge about patients' preferences, it was often related to eating position and the dining environment.

### Anticipation

Anticipating is when nurses ask questions or initiate talks about how the patients can be guided or helped to recover or when nurses take actions that promote the patients' physical, mental or emotional state [24]. Anticipation in nutritional care involves exploring the patients' dietary problems or initiating actions to promote and enhance their eating ability (Table 1).

The nurses described how they could promote the patients' appetite and desire to eat by creating a positive eating environment and serving the food delicately or serving small extra meals. To some nurses, these actions were essential for the patients' care and recovery. Nurses described how nutritional care was often based on the nurses' anticipation of what might be best for the patient rather than identifying the patient's needs in collaboration with the patient.

*We probably talk more about the patients between us nurses because I might have been taking care of Joan's patient for the past five days, and I may have noticed something …. So it's something you talk about with your colleagues. I think you could solve a lot of nutritional problems in that way. (Focus group 3)*

An ethical dilemma emerged amongst some nurses when patients had a deficiency in their dietary intake, and the nutritional guidelines recommend using a feeding tube in such cases. The dilemma originated from nurses' preferences, experiences and perceptions of whether a feeding tube would violate a patient's autonomy. Thus, the nurses reflected on offering the patient a feeding tube. The nurses, however, seldom involved the patients in their considerations and decisions to include a feeding tube or not.

*So, every time we have a patient, and we can see his nutritional needs are not met. Well, then we decide if he should get a feeding tube. (Focus group 2)*

When asked directly, the patients expressed that they would accept a feeding tube for recovery and survival. Overall, the patients expressed a need for nurses' support to improve their recovery process but rejected being told what to eat. Instead, they suggested that nurses provide different diet options. Especially the patients with low appetite asked nurses to engage in a dialogue to find the best ways to help them.

*I think it is important to talk about what you want to eat and have some choices. I don't feel pressured to eat. They ask me what I would like, and I think that is fine. (Patient 9)*

The observations showed positive interactions between nurses and patients when the nurse tempted the patient with new dishes or persuaded the patient to order dessert. The positive nurse–patient relationship was more often observed when nurses assisted patients in ordering dishes than when the nursing staff exclusively ordered the meals.

### Evaluating

Evaluating is when both patients and nurses review the patients' progress using feedback and negotiating the goals with others who need to be involved in the patient's rehabilitation [24]. Evaluating nutritional care is when nurses collaborate with the patients to evaluate the patients' nutritional needs by evaluating and adjusting goals within the nutritional plan (Table 1).

Nurses recognised the importance of monitoring the patients' dietary intake to evaluate their nutritional care. In clinical practice, serving meals was viewed as a simple service task that could be performed without knowing the patient. However, this approach was described as problematic because it made evaluating the patient's dietary intake over time difficult.

*Well, it's because we help each other deliver and fetch the trays with meals to the patients, and some students don't know that the note (describing the meal's nutritional content) should be saved, or we forget to save it ourselves when we serve meals. And then you don't notice it…. (Focus group 4)*

The organisation of the nutritional care in clinical practice maintained the evaluation of the nutritional care as a practical task. The practical approach did not support nutritional care as individual care, for example the night‐shift nurses were responsible for calculating and documenting the patients' daily dietary intake. The nurses described this as an impossible task and a waste of time.

When patients evaluated the nutritional care, many focused on the food delivery service. Although the patients appreciated the a la carte menu and were delighted with the variety of dishes, the physical environment did not stimulate their desire to eat. Some even described how they felt that nobody cared about whether they were eating or not:

*I do not know if anyone noticed (if he had eaten, eds). I did not say anything. I just told them that I had finished my meal, and when I couldn't eat it all, I just kept quiet. (Patient 1)*

The dietary intake was not consequently evaluated together with the patients. The observations showed that while some nurses accepted a low dietary intake, others encouraged the patients to try new food items or serve nutritional supplements by involving the patient.

*The nurse lifted the lid off the meal trays and notes significant leftovers. The nurses suggest other types of meals. They discuss possibilities, and patients agree to try a sip feed. (Field note)*

### Trusting

Trust is a prerequisite for establishing a relationship. Trust is formed when nurses exhibit behaviour that indicates the nurse's commitment to the patient's care. The concept of *trust* in nursing care involves developing a trusting relationship between the individual patient and nurse [24]. A trusting relationship can be established in nutritional care by respecting the patient's experiences and engaging in dialogue (Table 1)

The nurses rarely mentioned the importance of establishing a trusting relationship, while the patients more frequently talked about the lack of relationships. The lack of perceived alignment between their duty to care for the patients and the organisation's demands for documentation made the nurses struggle with a feeling of inadequacy.

*And sometimes, you don't have a choice, and you prioritise being present with the patient and forget about the rest. There are times when I suddenly realise, ‘Oh, my god. I forgot the (nutritional, eds.) screening. (Focus group 2)*

The patients trusted the nurses to know what was best for them. Some patients described themselves as experts in managing their disease and expected to be involved in their care. When the nurses fail to listen to patients´ nutritional concerns or needs, the patients experience a lack of trust.

The observations showed how nurses involved in serving the meals tried to create a pleasant eating environment for the patients but rarely used the short encounters during meal service to establish a patient relationship. The lack of attempt to establish a trusting relationship was observed when the nurses helped patients order their meals without asking them how they were doing.

## DISCUSSION

This study explored establishing of the nurse–patient relationship in nutritional care by using the FoC framework as an analytic framework. The findings show that the nurses´ capability to engage in dialogue was essential to establishing a nurse–patient relationship and a prerequisite for providing good nutritional care in the orthopaedic ward. A trusting relationship was established when the nurses were attentive and focused on the patients' individual needs by engaging in a nurse–patient dialogue.

The organisation of the nutritional care as a service task did not support the nurse–patient dialogue related to nutritional care. The nurses often failed to explore and include the patients' preferences and knowledge when making clinical decisions related to nutritional care, which decreased the patients' trust in the nurses.

We found the nursing staff focusing on serving meals and providing nutritional supplements without familiarising themselves with the orthopaedic patients' individual nutritional needs and preferences. Kitson et al. [24] describe that caring is more complex than just doing ‘things to people’, and Hestevik et al. [36] write that nutritional care goes beyond ensuring sufficient dietary intake [2]. When the food delivery routines shape the nurses' actions to be depersonalised and mechanistic, it may create a disharmonious interrelationship between nurses and the patients. The importance for patients to have a dialogue with the nurses must not be underestimated [37]. The patients in this study were not systematically involved in the decisions making regarding their nutritional care as the nursing staff perceived this as time‐consuming, which contrasts with the patients' call for nurses to respond to the patients' needs [38]. Communication that acknowledges the patient's role in care planning can make the difference in avoiding inadequate or missing care [39]. The patients in this study expressed feelings of safety when the nursing staff explored their nutritional needs. However, the patients expressed limited expectations towards the nurses as they believed eating was a personal matter and a part of their self‐care. Similarly, Kitson et al. [24] describe a tension between the ‘private complexity’ performed by a person as a self‐care task or activity and the ‘public simplicity’ performed by an institution, for example as nursing care in a hospital. When patients in this study asked for help to fulfil their nutritional needs and nurses failed to respond, some patients concluded that nutritional care was less important. This finding is supported by Avallin et al. [39] 2020 who describe how feelings of being unimportant will restrain patients from expressing needs, resulting in missed care.

To provide evidence‐based and high‐quality nutritional care, nurses must align their clinical practice with the recommendation of nutritional guidelines [13, 17]. The nurses in this study described feelings of inadequacy when prioritising spending time with the patient rather than complying with the documentation demands and the obligation to perform nutritional screening. One of the key recommendations from the guidelines is to provide an individualised nutritional plan to patients at risk of malnutrition [12]. The nurse's role is generally superficially described in these guidelines, which is problematic as nurses express a need for more knowledge to provide high‐quality nutritional care [9, 21, 40]. The results from this study highlight the relevance of including the FoC framework to provide a more comprehensive description of nursing care and actions within nutritional care. The FoC framework can be used to reflect, understand and operationalise what the guidelines describe as individualised nutritional care.

This study highlights the importance for nurses to anticipate the patient's nutritional challenges. Through patient dialogue, the nurses can secure sufficient and relevant knowledge to assess the patient's ability to eat and plan and perform high‐quality nutritional care. The FoC framework highlights the interaction between context and element of care and the dimensions of how care should be provided in combination with a compassionated approach to the patients in need of care [27]. As seen in this study, the patients' request for more dialogue is a clear statement to the nurses. The challenge for nurses is to combine evidence‐based knowledge with the nurse–patient relationship to provide good nutritional care that makes sense for both nurses and patients. When the nutritional care is organised and provided as task‐oriented, unrelated to the patient's individual needs and preferences, it does not promote high‐quality care. Focus on establishing the patients' trust in the nurses and making the nutritional guidelines relevant for nurses is necessary to improve clinical nutritional practice.

### Strength and limitation

The methodological strengths of this study are the use of three different data sources to perform a secondary analysis and the use of a well‐described framework as an analytic tool to identify the core dimension of the nurse–patient relationship. The use of the FoC framework may, however, at the same time be a limitation, as other elements of relevance could be overlooked. Using original data not collected with a focus on the elements of the FoC framework may result in the uneven distribution of codes in the elements. Further, the study's data represent one highly specialised setting, and the results may have a limited application. Finally, it added to the study's credibility that all authors worked closely with the analysis.

## CONCLUSION

The nurse–patient dialogue is a prerequisite for establishing a trustworthy relationship, and without such a relationship, nutritional care is at risk of being performed as a service task unrelated to the patients' needs. The patients' need for dialogue must not be compromised due to efficiency demands. More research is needed on how nurses establish the patient relationship in other clinical settings and how this shapes the quality of nutritional care.

## AUTHOR CONTRIBUTIONS

All authors contributed to the study design. PSJ and HVP contributed to the data collection. PSJ, VN and HVP contributed to the analysis and preparation of the manuscript. PSJ drafted the manuscript and all authors contributed to the critical revision of the manuscript and approved the final manuscript.

## ETHICAL APPROVAL

This study is a secondary analysis and includes data collected in previously approved studies. The Ethical Committee of The Capital Region of Denmark (H‐3‐2014_FSP67) reviewed the original studies regarding the patient and nurse interviews and considered not to require formal evaluation as the study design is not within the committee's scope. The Danish Data Protection Agency approved the original studies (2014‐041‐03395), and no further approval was needed for this secondary analysis. The observational data collection was approved by Danish Data Protection Agency (J.nr.: P‐2020‐540) as a part of a more extensive study on patients' experiences with nutritional care. All data included in this study were initially collected according to the Helsinki Declaration. All participants received oral and written information about the purpose of the original studies. The participants were informed about voluntary participation, anonymity, confidentiality, and the possibility to withdraw at any time. All participants signed a consent form before participating.

## CONFLICT OF INTEREST

The authors declared no conflict of interest with the respect to the research and authors and to the publication of this work and are alone responsible for the content and writing of the manuscript.
